# Neutrophil Extracellular Trap‐Related Gene Signatures and Molecular Clusters in Severe Influenza: Identification Through Integrative Transcriptome Analysis

**DOI:** 10.1155/jimr/6671885

**Published:** 2026-02-07

**Authors:** Libo Fei, Liang Chen

**Affiliations:** ^1^ Department of Critical Care and Emergency Medicine, The Second Affiliated Hospital of Nanjing University of Chinese Medicine, Nanjing, 210017, China, njucm.edu.cn; ^2^ Department of Infectious Diseases, Taikang Xianlin Drum Tower Hospital, Affiliated Hospital of Medical College of Nanjing University, Nanjing, 210046, China

**Keywords:** immunological characteristics, molecular clusters, neutrophil extracellular trap, severe influenza, transcriptome analysis

## Abstract

**Background:**

Neutrophil extracellular traps (NETs) are recently discovered structures in which neutrophils trap pathogens in web‐like structures composed of chromatin and proteolytic material. NETs have been linked to tissue damage in severe influenza (sFlu) pathogenesis. The present article involved a thorough analysis of *NET-related gene (NRG)* expression patterns and immunological characteristics in sFlu.

**Methods:**

Microarray datasets were downloaded from the GEO database. sFlu‐related NRGs (sFlu‐NRGs) were screened using differential expression analysis and weighted gene co‐expression network analysis (WGCNA). Hub sFlu‐NRGs were identified using LASSO regression, support vector machine (SVM), and random forest (RF) models. Hub genes were subsequently validated using an additional external dataset, clinical samples, and a nomogram model. The molecular clusters in sFlu were investigated based on the hub sFlu‐NRGs using consensus clustering and related immune cell infiltration.

**Results:**

A total of 13 sFlu‐NRGs were identified. Using these 13 genes, five (*PRTN3*, *MMP8*, *myeloperoxidase [MPO]*, *bactericidal permeability-increasing [BPI]*, *and LTF*) hub sFlu‐NRGs were identified using three machine learning algorithms. Nomogram calibration and receiver operating characteristic (ROC) analysis results suggested that accuracy was achieved in predicting sFlu. Two molecular clusters were defined in sFlu based on the five hub genes. Single‐set gene expression analyses suggested that, compared with Cluster 2, Cluster 1 had a decreased adaptive immune response.

**Conclusions:**

Five hub NRGs and two distinct NET‐related clusters were identified in sFlu patients, highlighting the mechanism of action of sFlu and identifying candidate anti‐sFlu therapeutic targets.

## 1. Introduction

Globally, influenza causes substantial annual illness and death, recurring as a widespread pandemic [1]. According to World Health Organization reports, seasonal influenza epidemics result in an estimated 3–5 million cases of severe illness worldwide each year, contributing to 290,000–650,000 respiratory‐related deaths [2]. Influenza can cause diverse clinical symptoms, ranging from a self‐limited upper respiratory tract infection to severe pneumonia. In critical cases of influenza, mortality rates can reach 50%–80%, and patients typically develop severe respiratory failure, manifested by an arterial oxygen pressure/fraction of inspired oxygen ratio <200 mmHg, requiring invasive mechanical ventilation (IMV) [3]. However, the pathogenic mechanism related to severe influenza (sFLu) remains unclear.

Neutrophils are the first innate immune cells recruited to infection sites and are associated with important effector mechanisms that control pathogen spread after influenza virus infection [4]. In addition to conventional mechanisms, neutrophils form DNA lattices called neutrophil extracellular traps (NETs), which entrap viruses and facilitate their killing [4]. The NET formation process is called NETosis, which is defined as a specific type of cell death characterized by the release of decondensed chromatin and granular contents into the extracellular space. The primary role of NETs is to prevent microbial transmission and severe infections. However, excessive NET formation has several disadvantages [4]. NETs have pathogenic effects on numerous human disorders, including influenza infection. DNA fibers within NETs can adhere to the capillary endothelium and are linked to vascular injury. Previous studies in mice revealed that excessive NET formation occurs in alveoli within tissue injury areas, which can increase the viral load and may subsequently progress to pathological signs similar to ARDS, such as diffuse alveolar injury, bleeding, hypoxemia, and pulmonary edema, indicating a possible relationship between NETs and lung injury [5]. According to the report of Zhu et al. [6], sFlu patients had higher plasma NET concentrations upon admission. NETs in H1N1 and H7N9 patients increased alveolar epithelial cell permeability, whereas NET generation was positively associated with MODS and the acute physiology and chronic health evaluation (APACHE) II score. Thus, NETs may be key predictors of poor prognosis in influenza patients. Nonetheless, to date, thorough research on NETs and sFlu is lacking.

**Figure 1:**
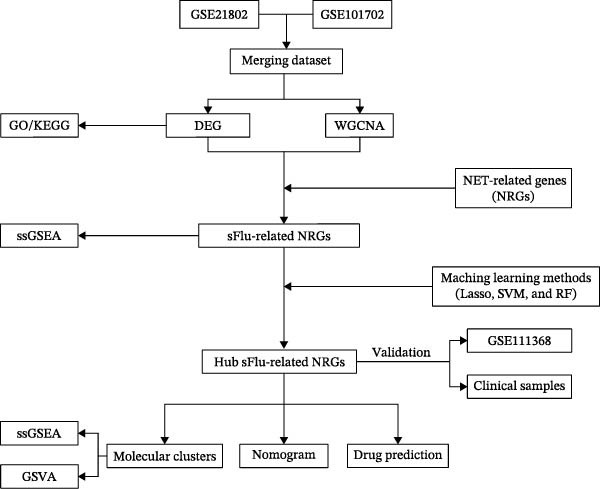
Study flowchart.

This article, therefore, aimed to determine the relationship between sFlu and NRGs. First, the influenza datasets were downloaded from the GEO database, and NRGs were obtained from the literature. The sFlu‐related NRGs (sFlu‐NRGs) were screened using differential expression analysis and weighted gene co‐expression network analysis (WGCNA). Hub sFlu‐NRGs were identified using least absolute shrinkage and selection operator (Lasso) regression, support vector machine (SVM), and random forest (RF) models based on these hub genes. Nomogram and receiver operating characteristic (ROC) analyses were conducted to assess the diagnostic value of the hub genes in patients with sFlu. The patients with sFlu were subsequently assigned to two clusters based on the expression profiles of the hub sFlu‐NRGs. Changes in cluster‐specific gene signaling pathways and associated immune cell infiltration were evaluated using GSVA and ssGSEA, respectively (Figure 1).

## 2. Materials and Methods

### 2.1. Data Collection

The mRNA expression data derived from the blood samples of influenza patients were retrieved from NCBI‐GEO (http://www.ncbi.nlm.nih.gov/geo). The screening criteria were as follows: (i) influenza infection verified through reverse transcription polymerase chain reaction (RT–PCR) using respiratory tract specimens; (ii) influenza cases of patients ≥16 years old; and (iii) the same disease severity. The definition of sFlu was influenza patients with respiratory failure who needed IMV. Influenza cases not requiring mechanical ventilation served as the control group. Three datasets, namely, GSE21802 (*N*_sFlu_ = 20, *N*_non-sFlu_ = 16), GSE111368 (*N*_sFlu_ = 69, *N*_non-sFlu_ = 160), and GSE101702 (*N*_sFlu_ = 44, *N*_non-sFlu_ = 63), were obtained. The GSE111368 and GSE21802 datasets were pooled into the training dataset. The R “sva” package (v. 4.4.0) was utilized to eliminate batch effects, and separate dataset levels were log_2_‐transformed before cross‐platform normalization. Next, gene levels across diverse samples or databases were normalized using the ComBat approach, and batch effects were removed via principal component (PC) analysis. In addition, the GSE101702 dataset was used as the test dataset to verify the hub genes. A total of 573 NRGs (Supporting Information 1) were identified in the current article and obtained from prior studies [7], and the public website GeneCards (https://www.genecards.org/).

### 2.2. Selection of Differentially Expressed Genes (DEGs)

The “limma” package (v.4.4.1) was used to identify DEGs between patients with sFlu and non‐sFlu, and *p* *<*0.05 and |log_2_FC| > 1 were used as screening criteria. A volcano plot was used to display the resulting data, and a heatmap was used to present the 50 most significant DEGs.

### 2.3. WGCNA

Using the “WGCNA” package (v.4.4.1) in R, the gene expression patterns in the training set were obtained through WGCNA. The genes showing the top 30% absolute deviation from the median were subsequently chosen. Data accuracy was ensured using the “goodSampleGenes” function. Using the “pickSoftThreshold” function, the optimal soft threshold (*β*) was selected and validated. The matrix data were later incorporated into the adjacency matrix, and modules were identified through clustering based on topological overlap. When the module eigengene (ME) was computed while the relevant modules from the ME‐based clustering tree were combined, a hierarchical clustering dendrogram was obtained. The phenotypic data and modules were subsequently used to assess module significance (MS) and gene significance (GS), and to identify GS and clinical data while determining the relationships between the modules and the models. Moreover, the module membership (MM) for each gene was computed to determine the module’s GS.

### 2.4. Identification of sFlu‐NRGs and Functional Enrichment Analysis

The sFlu‐NRGs were obtained through the intersection of DEGs, NRGs, and module‐related genes from WGCNA. The R package “cluster profile” (v.4.4.1) was used for GO annotation and KEGG analysis. GO terms and KEGG pathways were screened based on *p*  < 0.05. Biological process (BP), cellular component (CC), and molecular function (MF) were the three GO term categories.

### 2.5. Identification of Hub sFlu‐NRGs Using Machine Learning Methods

Three machine learning algorithms, namely, LASSO, SVM, and RF, were employed to further filter the candidate hub *sFlu-NRG* genes. LASSO regression uses a penalty on absolute coefficient values to regulate their magnitudes, thereby improving control over overfitting and increasing model interpretability [8]. In addition, the SVM–RFE model is used to identify key disease‐related signature genes by removing redundant and noisy features, thereby reducing data dimensionality while enhancing model accuracy [9]. The RF algorithm allows the construction of decision trees from random data and feature subsets, thereby reducing tree associations and improving overall performance [10].

### 2.6. Immune Cell Infiltration

Using the ssGSEA algorithm, immune cell infiltration levels in the training set samples were analyzed. Additionally, violin plots were generated to visualize the differential expression of infiltrating immune cells. Moreover, Spearman correlations between infiltrating immune cells and genes were determined using the “ggplot2” package (v.4.4.3).

### 2.7. Differential Analysis and ROC Curve Verification

The “limma” (v.4.4.1) and “ggpubr” (v.4.4.1) functions in R were used to analyze the differential expression of these hub genes between the sFlu and non‐sFlu groups. ROC curves were drawn for the hub genes using the R package “pROC” (v.4.4.1), and the area under the curve (AUC) and 95% confidence interval (CI) were computed. Typically, an AUC >0.7 suggests optimal predictive performance.

### 2.8. Nomogram Model Construction and Verification

Using the hub sFlu‐NRGs, “rms” (v.4.4.3) in R was utilized for nomogram construction. “Points” suggests a candidate gene score, whereas “Total Points” represents the sum of these aforementioned scores. A combination of DCA and a calibration curve was subsequently used to estimate the nomogram’s predictive performance.

### 2.9. Unsupervised Clustering of sFlu Cases

Based on the hub’s sFlu‐NRG expression patterns, the R “ConsensusClusterPlus” package (v.4.4.0) was used for unsupervised clustering and classification of sFlu cases into distinct clusters using the k‐means algorithm (1000 iterations). On the basis of the consistent cluster score (>0.8), cumulative distribution function (CDF) curve, and consensus matrix, the greatest cluster k value (i.e., 9) was selected, and the best number of clusters was determined.

### 2.10. GSVA

GSVA was performed using the R “GSVA” package (v. 4.4.0) to evaluate gene expression changes across diverse clusters. The “c2.cp.kegg.symbols” from the MSigDB database was utilized for additional GSVA evaluation. Furthermore, the “limma” package (v.4.4.1) in R was used to assess changes in biological functions and pathways by analyzing GSVA scores across diverse clusters, using *p*‐value thresholds <0.05 and t‐value thresholds >2.

### 2.11. Drug Prediction

Using the hub sFlu‐NRGs, the targeted drug molecule was prepared using the Drug Signatures database (DSigDB, https://amp.pharm.mssm.edu/Enrichr/). Later, Enrichr, an enrichment analysis platform that contains wide‐spectrum visualization data, was utilized to assess the collective functions of genes.

### 2.12. qRT–PCR

The RNA from 10 paired samples was extracted using TRIzol (Life Technologies, CA, USA) and subsequently transformed into cDNA using PrimeScript RT Master Mix (Takara, Tokyo, Japan). Afterward, qPCR was performed on an ABI 7700 system (Applied Biosystems, CA, USA), and β‐actin was used as a reference. The 2^–ΔΔCt^ method was used to assess gene expression. The utilized qPCR primer sequences are provided in Supporting Information 2.

### 2.13. Statistical Analysis

R (v. 4.4.1) was used for the statistical analysis. Normally distributed data were evaluated using Student’s *t* test. Nonnormally distributed data were evaluated using the Mann–Whitney test. Pearson correlation and Spearman correlation analyses were conducted to determine the correlations.  ^∗^ *p*  < 0.05,  ^∗∗^ *p*  < 0.01, and  ^∗∗∗^ *p*  < 0.001 indicate statistical significance.

## 3. Results

### 3.1. Cross‐Platform Normalization

First, the GSE111368 and GSE21802 datasets were combined into a single training dataset, yielding 11,116 genes. Before batch effects were eliminated, these datasets exhibited batch‐based clustering along the two top PC axes generated from the nonnormalized gene‐level data (Supporting Information 6: Figure S1A). PC analysis, conducted using normalization, verified the successful removal of batch effects (Supporting Information 6: Figure S1B), demonstrating cross‐platform normalization.

### 3.2. Identification of DEGs and Functional Enrichment Analysis

A total of 43 DEGs were identified in sFlu patients compared with non‐sFlu patients, including five downregulated DEGs and 37 upregulated DEGs (Supporting Information 6: Figure S2A,B and Supporting Information 3).

According to the GO annotation results, the DEGs were associated with immunity BPs, including defense response to bacteria, response to molecules of bacterial origin, and response to lipopolysaccharides (LPS, Supporting Information 6: Figure S2C and Supporting Information 4). According to the KEGG results, the enriched pathways included antigen processing and presentation, phagosome, and graft‐versus‐host disease (Supporting Information 6: Figure S2D and Supporting Information 4).

### 3.3. WGCNA

WGCNA was used to identify key modules associated with sFlu by constructing a co‐expression network from the training set. Hub gene levels and diagnostic performance were evaluated, with a soft‐thresholding power = 14 (scale‐free *R* ^2^ = 0.9) and cutoff height = 0.30 (Figure 2A,B). The scope of the 10 modules was subsequently reduced (Figure 2C). The relationships of sample features to ME values were utilized to measure and assess the data via a heatmap, where the magenta module had the closest relationship with disease severity (cor = 0.46, *p* = 2e–15) (Figure 2C).

Figure 2WGCNA and the discovery of hub sFlu‐NRGs. (A) Scale‐free fit index and mean connectivity analyses under diverse soft‐thresholding powers. The red line indicates a correlation coefficient of 0.9, whereas the soft‐thresholding power (*β*) is 14. (B) Gene dendrograms with the dissimilarity metric (1‐TOM, the red line represents the cutoff height = 0.3). (C) Module–trait associations of module eigengenes with sample traits were evaluated. The correlation coefficient and the *p*‐value were obtained for every cell. (D) Venn diagram presenting the intersections of DEGs, NRGs, and module‐related genes.

**Figure:**
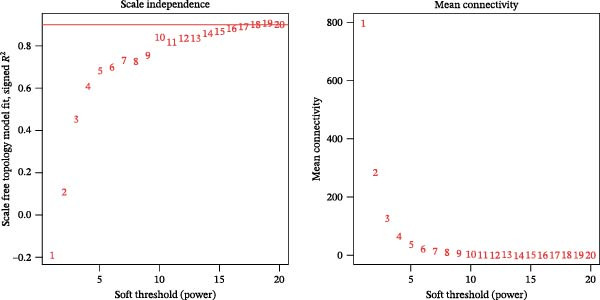
(A)

**Figure:**
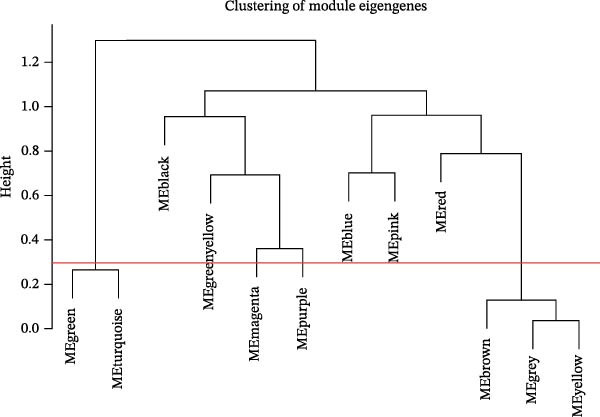
(B)

**Figure:**
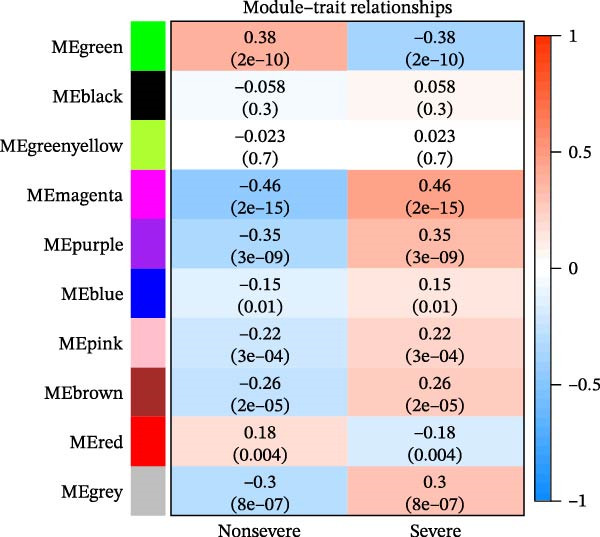
(C)

**Figure:**
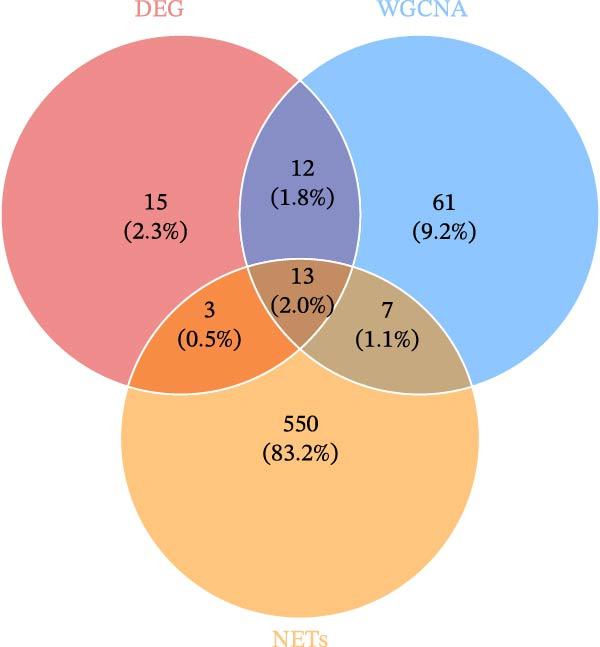
(D)

### 3.4. Identification of sFlu‐NRGs and ICI Analysis

The DEGs, NRGs, and magenta module genes were intersected, revealing 13 overlapping genes, which were identified as sFlu‐NRGs (Figure 2D). All of these genes were upregulated in the sFlu group compared with the non‐sFlu group (Supporting Information 6: Figure S3A).

The ssGSEA algorithm was applied to evaluate differences in ICI efficacy between sFlu and non‐sFlu patients (Supporting Information 5). The ssGSEA results revealed significantly lower levels of adaptive immune cells (e.g., activated CD8^+^ T cells, activated B cells, memory CD4^+^ T cells, memory B cells, and memory CD8^+^ T cells) and greater levels of innate or inflammatory cells (e.g., macrophages, activated dendritic cells, mast cells, etc.) in the sFlu group than in the non‐sFlu group (Supporting Information 6: Figure S3B). Immunological cell correlation analysis revealed that the 13 sFlu‐NRGs were positively associated with inflammatory cells but negatively related to adaptive immune cells (Supporting Information 6: Figure S3C).

### 3.5. Identification of Hub sFlu‐NRGs Using Machine Learning Methods

The SVM‐RFE (Figure 3A,B), LASSO (Figure 3C,D) and RF (Figure 3E,F) results were pooled to determine the key NRGs related to sFlu. The three methods revealed 7, 6, and 13 important NRGs, respectively. Eventually, 5 overlapping NRGs (*PRTN3*, *MMP8*, *MPO*, *BPI*, and *LTF*) were selected as hub sFlu‐NRGs (Figure 4A).

Figure 3Machine learning algorithms used to screen the candidate key sFlu‐NRGs. (A,B) Possible hub genes identified through LASSO regression under 10‐fold cross‐validation. (C,D) Representative NRGs (*n* = 6) obtained via SVM–RFE algorithm‐based identification. (E) The plot shows the random forest tree, in which the *x*‐axis represents the tree number and the *y*‐axis represents the error rate. The red, green, and black dots represent the sFlu, non‐sFlu, and overall samples, respectively. (F) Horizontal and vertical axes represent gene importance and NRGs, respectively.

**Figure:**
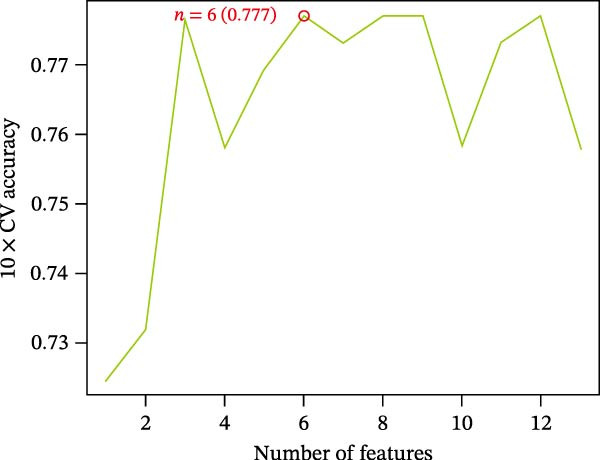
(A)

**Figure:**
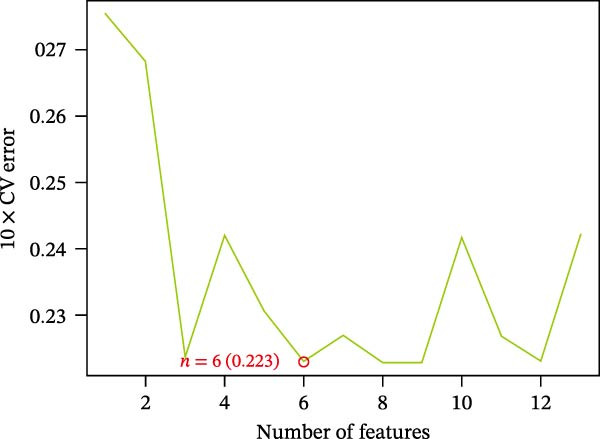
(B)

**Figure:**
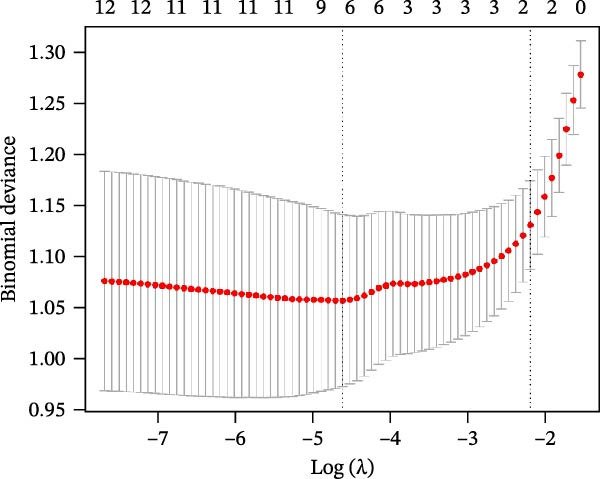
(C)

**Figure:**
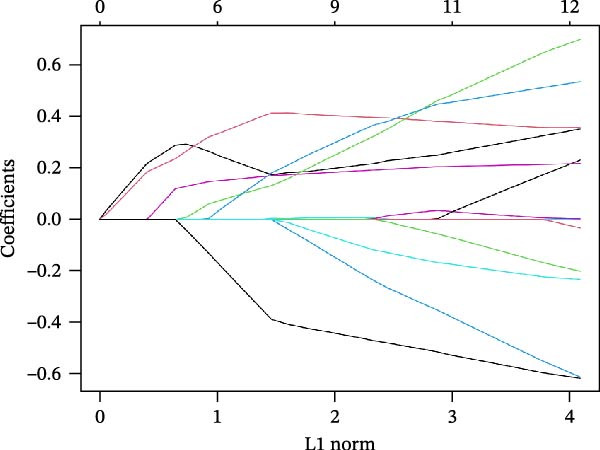
(D)

**Figure:**
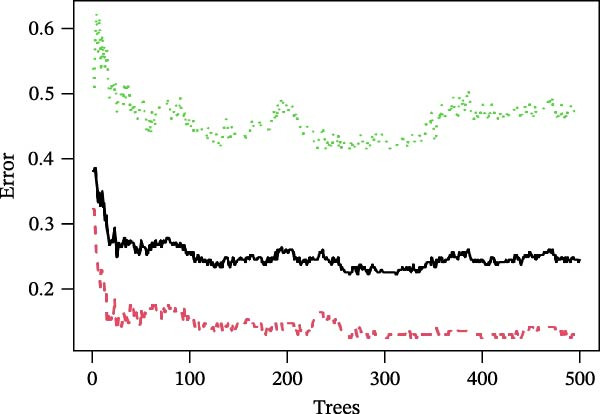
(E)

**Figure:**
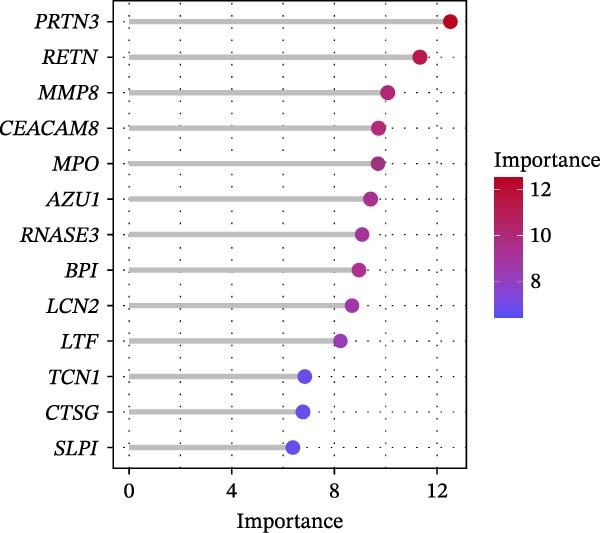
(F)

Figure 4Identification of the hub sFlu‐NRGs and their verification using the training set. (A) Diagram showing the five hub genes identified using the three machine learning methods. (B) Boxplots were drawn to validate the expression of the five hub NRGs in the training set.  ^∗∗∗^ *p*  < 0.001. (C) ROC analysis of the training set was conducted to validate the diagnostic performance of the hub NRGs. (D) ROC analysis of the training set was conducted to validate the diagnostic performance of the five‐gene logistic regression model.

**Figure:**
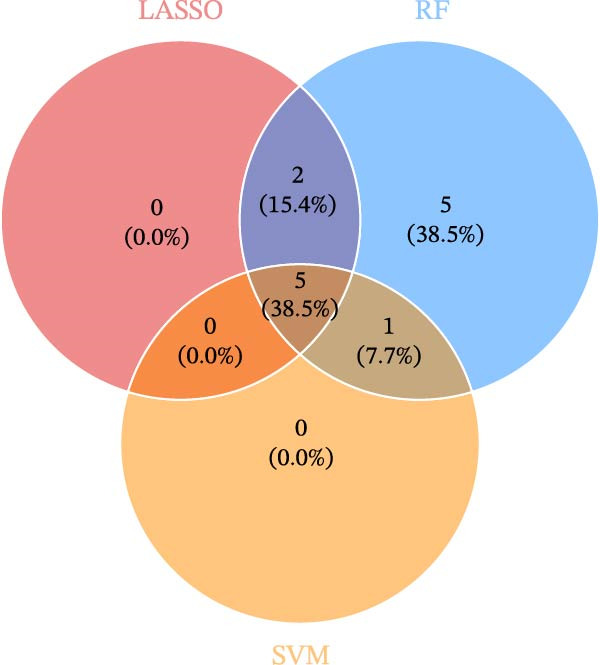
(A)

**Figure:**
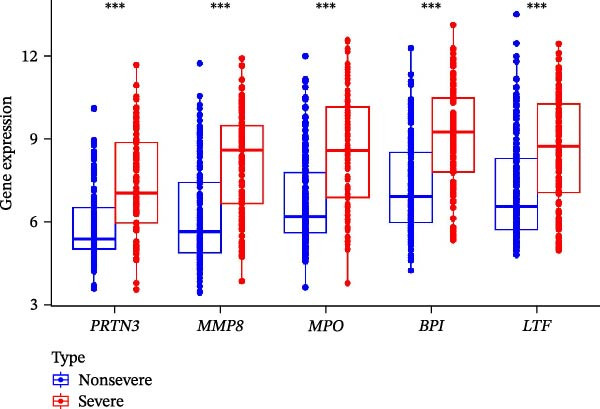
(B)

**Figure:**
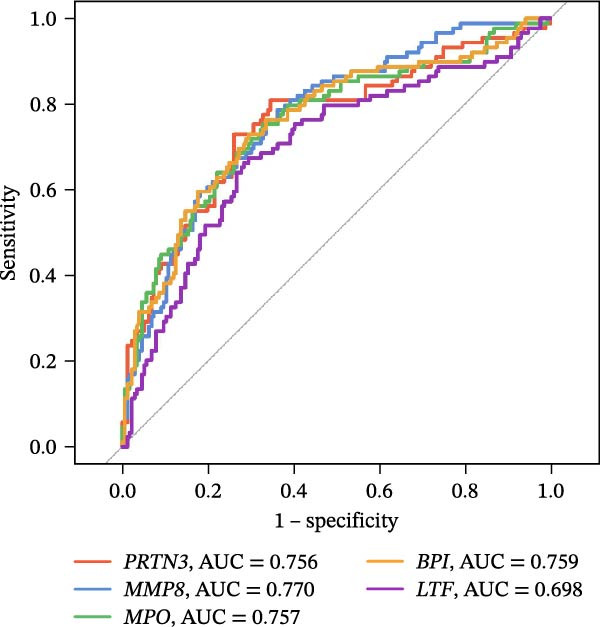
(C)

**Figure:**
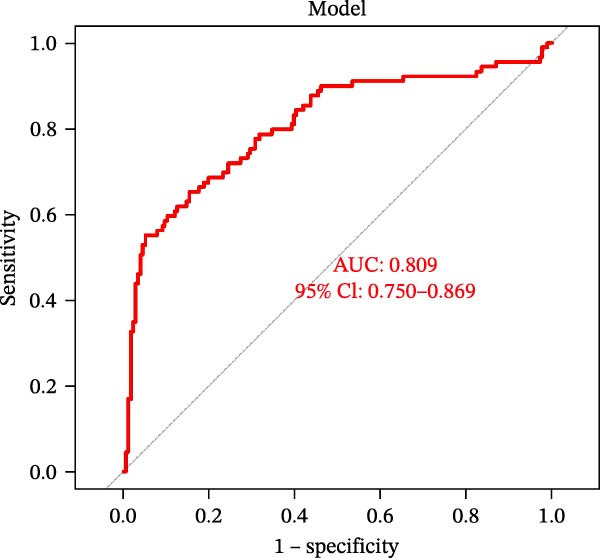
(D)

The expression levels of these five hub genes were determined using boxplots. As shown in Figure 4B, patients with sFlu in the training set had higher hub gene levels than non‐sFlu patients. The same findings were obtained in the testing set GSE101702 (Figure 5A) and clinical samples (Figure 5D). The ROC analysis of the training set revealed that, with the exception of TLF (AUC = 0.698), the AUCs of all the other (four) hub genes ranged from 0.756 to 0.770 (Figure 4C). The AUC of the five‐gene logistic regression model was 0.809 (Figure 4D). Moreover, the AUCs of the five hub genes and the five‐gene logistic regression model were above 0.7 in the testing set, suggesting ideal diagnostic accuracy (Figure 5B,C).

Figure 5Confirmation of the hub NRGs in the testing set and clinical samples. (A) Boxplots were constructed to validate the expression of the five hub NRGs in the testing set. (B) ROC analysis of the testing set was utilized to validate the diagnostic performance of the five hub NRGs. (C) ROC analysis of the testing set was adopted to validate the diagnostic utility of the five‐gene logistic regression model. (D) Boxplots were constructed to validate the expression of the five hub NRGs in clinical samples.  ^∗∗∗^ *p*  < 0.001.

**Figure:**
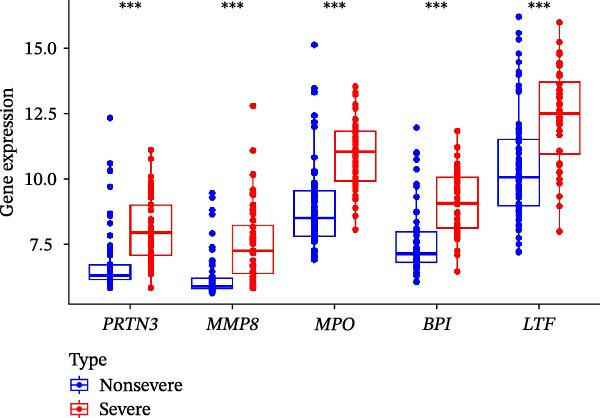
(A)

**Figure:**
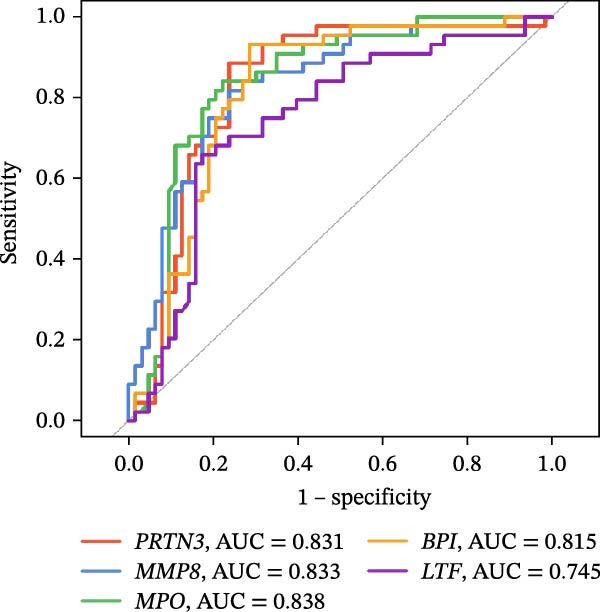
(B)

**Figure:**
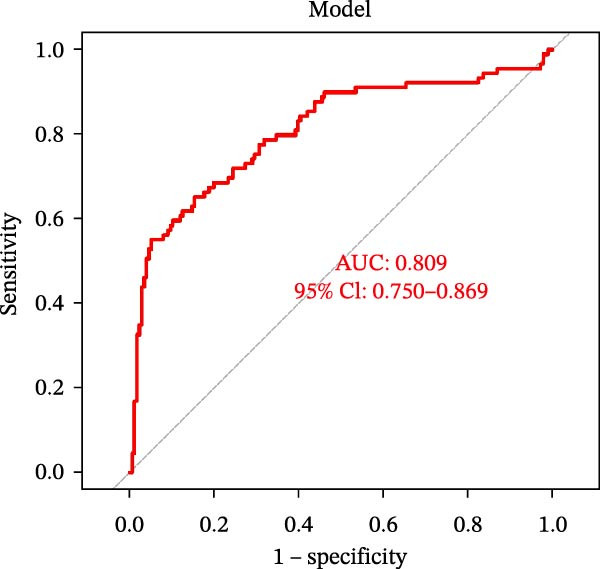
(C)

**Figure:**
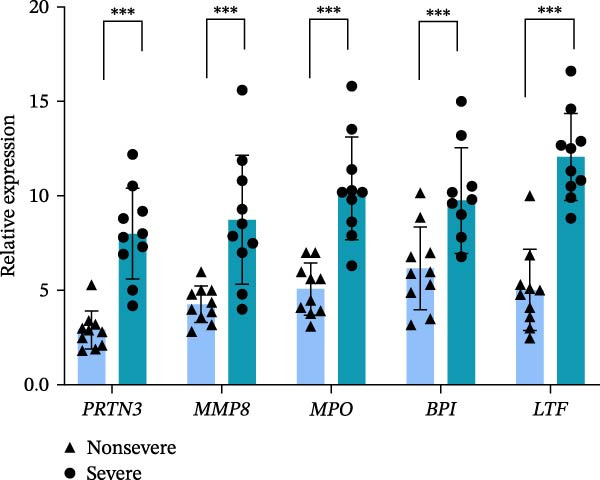
(D)

### 3.6. NET‐Related Nomogram Model Construction and Verification

Based on the 5 hub NRGs, a NET‐related nomogram was constructed to serve as a streamlined, reliable diagnostic tool for sFlu (Figure 6A). According to the calibration curve, the error between the real and predicted risks was quite low (Figure 6B), whereas DCA showed the high accuracy of the constructed nomogram (Figure 6C).

Figure 6Nomogram model construction based on the five hub NRGs. (A) Nomogram showing the diagnostic significance of the five hub NRGs for sFlu patients. (B) Calibration curve for assessing the predictive ability of the nomogram. (C) DCA curve for evaluating the utility of the nomogram.

**Figure:**
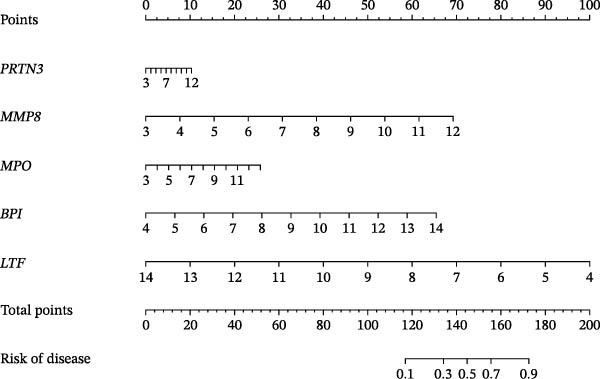
(A)

**Figure:**
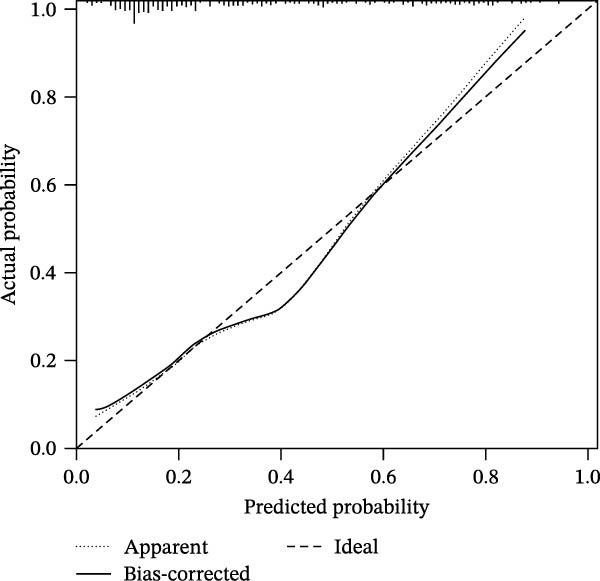
(B)

**Figure:**
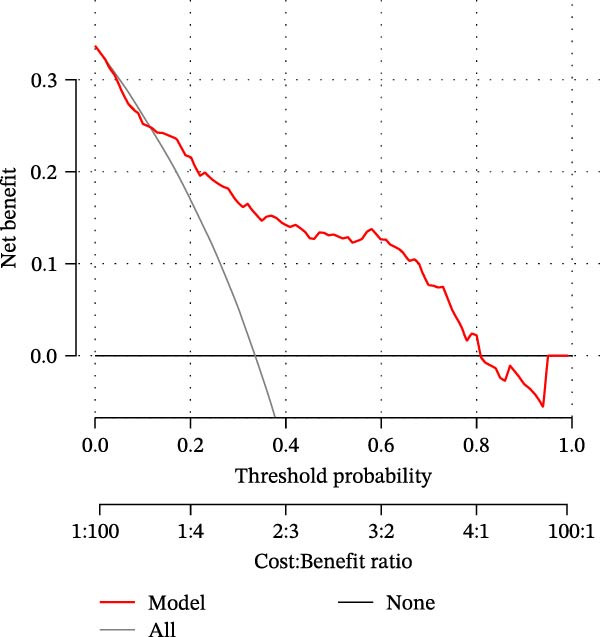
(C)

### 3.7. Unsupervised Clustering for sFlu Cases

Based on the five hub gene levels, the consensus clustering approach was used to classify the 89 sFlu patients in the training set, thereby determining NRG expression patterns in sFlu. At *k* = 2, the most stable clusters were obtained (Supporting Information 6: Figure S4A). At the consensus index = 0.2–0.8, the CDF curves could be distinguished in the minimal range (Supporting Information 6: Figure S4B). At *k* = 2–9, the AUC values of the CDF curves suggested changes in two CDF curves (*k* and *k* − 1) (Supporting Information 6: Figure S4C). In addition, t‐distributed stochastic neighbor embedding analysis revealed clear differences between two clusters (Supporting Information 6: Figure S4D).

### 3.8. NET Regulators, Immune Infiltration Features, and Functional Annotation Comparisons Among the NET Clusters

Hub gene expression levels were analyzed in the two clusters. As shown in Figure 7A,B, all five hub genes were upregulated in Cluster 1 compared with Cluster 2.

Figure 7NET regulators, immune infiltration features, and functional associations among NET clusters. (A) Boxplots showing the five hub NRG levels in two clusters. (B) Heatmap showing the five hub NRG levels in two clusters. (C) Differential hallmark pathway activity in Cluster 1 versus Cluster 2 samples ranked based on t value using GSVA. (D) Boxplots showing the differential immune cell infiltration in the 2 clusters.  ^∗^ *p*  < 0.05,  ^∗∗^ *p*  < 0.01,  ^∗∗∗^ *p*  < 0.001.

**Figure:**
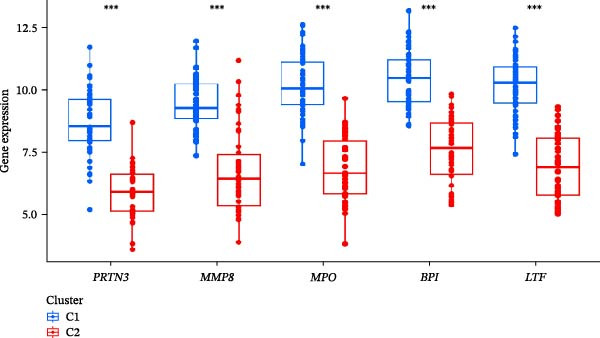
(A)

**Figure:**
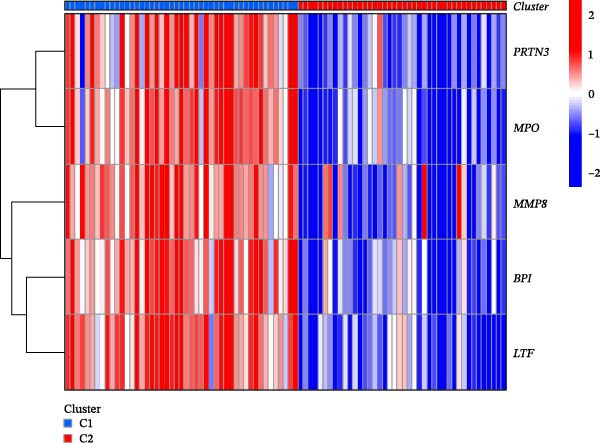
(B)

**Figure:**
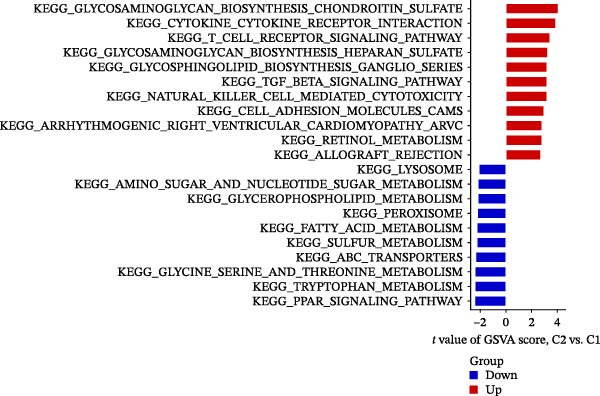
(C)

**Figure:**
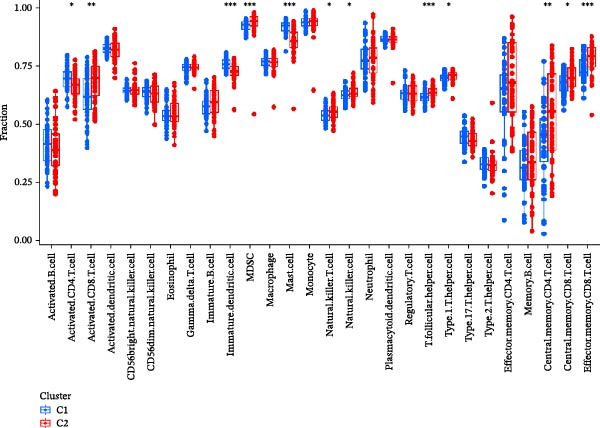
(D)

GSVA revealed that some pathways regulating immune responses (e.g., KEGG_CYTOKINE_CYTOKINE_CYTOKINE_RECEPTOR_INTERACTION, KEGG_T_CELL_RECEPTOR_SIGNALING_PATHWAY, and KEGG_NATURAL_KILLER_CELL_MEDIATED_CYTOTOXICITY) were downregulated in Cluster 1, whereas the pathways related to the metabolism of amino acids, carbohydrates, and fats (e.g., KEGG_TRYPTOPHAN_METABOLISM, KEGG_GLYCINE_SERINE_AND_THREONINE_METABOLISM, KEGG_FATTY_ACID_METABOLISM, and KEGG_GLYCEROPHOSPHOLIPID_METABOLISM) were upregulated in Cluster 1 (Figure 7C).

According to the results of immune infiltration analyses, some adaptive immune cell traits (such as type 1T helper cells, activated CD8^+^ T cells, central memory CD4^+^ T cells, natural killer T cells, and memory CD8^+^ T cells) exhibited decreased infiltration in Cluster 1, whereas mast cells exhibited increased infiltration in Cluster 1 (Figure 7D).

### 3.9. Predicting HubsFlu‐NRGs That Target Drugs

Drugs that efficiently target the hub sFlu‐NRGs were identified in the DGIdb database, and the results were visualized in Cytoscape (Supporting Information 6: Figure S5). Ten drugs that target *MPO*, *LTF*, *PRTN3*, and *MMP8* were identified, among which only one drug could impact *BPI* (Supporting Information 6: Figure S5).

## 4. Discussion

Although medical science has been progressing continuously, sFlu remains a major burden on public health, causing hospitalization and deaths worldwide [1–3]. Acute pneumonia following influenza virus infection is characterized by excessive neutrophil infiltration into the lungs [11]. Activated neutrophil‐released NETs aggravate inflammation, and the NETs produced recruit neutrophils, creating a feedforward cycle [4, 11]. Although an increasing number of studies have shown that NETs are related to sFlu pathogenesis, the detailed mechanisms remain unknown. The latest transcriptomic studies have highlighted the involvement of various genes and gene levels related to influenza‐related disease severity [12]. However, to the best of the author’s knowledge, this study is the first to systematically explore NRGs in patients with sFlu using multiple datasets and integrative bioinformatics tools.

In the last 20 years, computational applications with high effectiveness and accuracy have been developed to utilize most biomedical data resources [13]. Among bioinformatics approaches, WGCNA is advantageous because it focuses on the relationships between clinical features and co‐expression modules, thereby yielding comprehensive, highly reliable, and biologically significant data [14]. The integrated use of WGCNA and differential expression analysis in this study identified 13 NRGs closely associated with influenza severity. Then, LASSO, SVM, and RF machine‐learning classifiers based on these 13 NRG expression patterns were used to identify the core sFlu‐NRGs. Machine learning algorithms offer several advantages, including enhanced model accuracy, improved interpretability, reduced overfitting, and efficient processing of high‐dimensional data [15]. In biomedical studies, the key modules related to disease processes can be precisely predicted, and the possible clinical features can be determined. In this study, five NRGs were ultimately identified as key sFlu‐NRGs, which demonstrated accurate performance in predicting sFlu. Moreover, a nomogram was constructed using five hub genes, providing a simple and reliable tool to facilitate clinical risk assessment for clinicians involved in the management of influenza patients.

Immune responses have important effects on the pathogenesis and resolution of influenza infection [11, 16]. In this study, the ssGSEA results demonstrated that immune cell infiltration significantly differed between sFlu patients and non‐sFlu patients. One involves the suppression of adaptive immune cells, and the other involves the activation of innate or inflammatory cells. Numerous studies have reported significant lymphopenia in sFlu, possibly due to apoptosis, disruption of antigen presentation, abortive infection of primary human T cells, and T‐cell exhaustion related to cytokines and viruses [17–19]. The suppression of adaptive immune responses delays viral clearance. However, excessive infiltration of inflammatory cells is linked to tissue injury [11, 20]. In this study, all sFlu‐NRGs were upregulated in the sFlu group and were positively associated with inflammatory cells but negatively related to adaptive immune cells. NRG dysregulation and its relationship with immune cells suggest that NRGs may participate in the immunological pathogenesis underlying sFlu. Based on the expression patterns of the five hub NRGs, the unsupervised clustering conducted in this study revealed distinct NET regulatory patterns among sFlu individuals, yielding two clusters. On the basis of the subsequent ICI analysis, Cluster 1 exhibited the typical feature of suppressive adaptive immunity. GSVA of the cluster‐specific NRGs revealed that the pathways regulating immune responses were downregulated in Cluster 1, suggesting that Cluster 1 was closely related to sFlu immunopathology.

The *PRTN3* gene encodes proteinase 3 (PR3), a member of the neutrophil serine protease family, which can be produced by activated cells and is important for ARDS pathophysiology, as revealed in preclinical and clinical trials [21]. PR3 plays a key role in neutrophil‐mediated killing of microorganisms, and neutrophil activation facilitates cytokine production, leading to lung injury [22]. Bayat et al. [23] reported that PR3, in complex with the soluble neutrophil surface protein CD177, present in the supernatant of packed red blood cells, specifically binds to endothelium, inducing reactive oxygen species (ROS) generation and leading to transfusion‐related acute lung injury. Exposure of sCD177/PR3‐positive endothelial cells to anti‐PR3 antibodies triggers endothelial activation, increased permeability, and severe barrier impairment.

Matrix metalloproteinase‐8 (MMP8), also referred to as neutrophil collagenase or collagenase 2, is stored within neutrophil granules as an inactive enzyme and is rapidly released into inflammation sites when neutrophils are activated [24]. Owing to its characteristic features, MMP8 plays a key role in the pathogenic mechanism underlying acute respiratory distress syndrome or acute lung injury [25]. MMP8 plays the most important role in degrading collagen type I, the main extracellular matrix component of the lung. In addition, MMP8 is highly sensitive to ROS, which are usually related to lung diseases. Lin et al. [26] reported that in patients with H1N1 influenza, MMP8 and PRTN3 were upregulated and associated with increased disease severity. MMP8 modulates macrophage and neutrophil accumulation within lung tissues in LPS‐mediated lung injury [27].

As a heme‐containing coenzyme, myeloperoxidase (MPO) belongs to the heme peroxidase superfamily [28] and generates ROS and hypochlorous acid (HOCl) using hydrogen peroxide (H_2_O_2_) and chloride ions as substrates [29]. MPO has been reported to exacerbate disease in an influenza virus infection mouse model when MPO‐ROS‐producing enzymes are used [29]. As reported by De La et al. [30], ROS produced by the influenza A virus, pro‐inflammatory factors, and cell mortality are strongly associated with increased MPO content. After viral NS1 plasmid transfection, more MCP‐1 and IL‐8 were produced in the cells treated with the H_2_O_2_‐MPO system. The serum MPO content is markedly increased in H5N1‐ and ARDS‐infected patients, and MPO has been identified as an independent risk factor for predicting severe H1N1 influenza [31].

Neutrophil lactoferrin (LTF) has diverse effects, including antibacterial, anti‐inflammatory, anticancer, and immunomodulatory activities [32]. In acidic environments, such as during pepsin digestion, lactoferrin generates small‐molecular‐weight antibacterial peptides that inhibit bacteria and modulate inflammation in different ways. In mice, lactoferrin administration significantly counteracted carfilzomib‐induced inflammation in the lungs by inhibiting the NLRP3/NF‐κB and PI3K/Akt/GSK‐3β/MAPK axes [33]. Lactoferrin feeding in H5N1 influenza virus‐infected mice mitigated vascular congestion and dilatation, decreased inflammatory cell infiltration, and improved alveolar septal widening [34]. Since the cationic protein separated from human neutrophils is related to specific neutrophil granules [35], bactericidal permeability‐increasing (BPI) protein can bind to LPS, neutralize the effects of LPS, and mitigate the effects of endotoxins [36]. Previously, recombinant BPI therapy was shown to suppress endotoxin‐mediated activation of circulating neutrophils and to inhibit the generation of inflammatory factors, thereby mitigating acute lung injury [37]. LTF and BPI are generally more abundant in relatively sFlu cases, possibly exerting a protective role.

Certain limitations of the present article must be noted. First, efforts were made to identify relevant public datasets, but the study’s sample size was small, which may have introduced bias. Many large‐scale studies using advanced sequencing technologies and constructing high‐accuracy nomograms have combined these models with additional data (such as clinical parameters). Second, the relationships between NRGs and infiltrating immune cells must be categorized as statistical correlations without causality. Similarly, whether such host variables are specific to sFlu infection or just to ventilation remains unclear. Confounding factors, such as mixed or secondary bacterial infection potentially necessitating mechanical ventilation, could influence the results. However, sFlu requiring IMV is a unique clinical phenotype that also encompasses secondary bacterial infections, and this conclusion should not be undermined when it is studied as an integrated whole. Finally, microarrays are associated with several disadvantages (e.g., nonwhole‐genome analysis, high background signal levels, lack of quantity, and inability to detect alternative splicing). Therefore, more in vitro and in vivo assays that assess the effects of these NRGs should be conducted to elucidate the pathogenic mechanisms of sFlu.

## 5. Conclusion

Overall, the expression of NRGs in patients with sFlu was comprehensively evaluated. Five hub NRGs were identified using integrated bioinformatic methods. Based on these hub genes, a nomogram model was constructed to accurately predict sFlu risk, along with immune cell infiltration analysis, molecular clustering, and gene‐targeted drug prediction. This article highlights the benefits of using sFlu at the molecular and immune scales. The clinically used drugs to treat influenza target viral replication; however, there is a lack of medications that modulate the immune response specifically for sFlu. We screened key NRGs that play critical roles in sFlu pathogenesis, along with molecules that target these genes. These findings suggest potential therapeutic targets and drug candidates for the future treatment of sFlu. Moreover, the NET‐associated signatures revealed in this article must be verified using laboratory data.

## Author Contributions

**Libo Fei**: design, data collection, data analysis, manuscript writing, manuscript revision. **Liang Chen**: conception, design, data collection, data analysis, funding acquisition, manuscript writing, manuscript revision, and supervision.

## Funding

The study was supported by the Nanjing Medical Science and Technology Development Fund (Grant YKK22239).

## Disclosure

All the authors have approved the final manuscript.

## Conflicts of Interest

The authors declare no conflicts of interest.

## Supporting Information

Additional supporting information can be found online in the Supporting Information section.

## Supporting information


**Supporting Information 1** List of 573 NRGs.

**Supporting Information 2** qPCR primer sequences.

**Supporting Information 3** List of 43 DEGs between patients with sFlu and non‐sFlu.

**Supporting Information 4** Detailed results of the GO and KEGG analyses.

**Supporting Information 5** Detailed results of ssGSEA.

**Supporting Information 6** Figure S1: Principal component analysis of the gene expression dataset. The dots in the scatter plot are based on the first two main components of the gene expression profile (PC1 and PC2) visualization samples: (A) no elimination of the batch effect; (B) after elimination of the batch effect. The colors represent samples from the two different datasets. Figure S2: Gene expression profiling and functional enrichment analysis in the training set. (A) Heatmap of the top 50 DEGs. The upregulated genes are shown in red, whereas the downregulated genes are highlighted in blue. (B) DEG volcanic plot. The upregulated genes are highlighted in red, whereas the downregulated genes are indicated in blue. GO (C) and KEGG (D) analyses of the DEGs. Figure S3: Expression profiling and ICI analysis of 13 sFlu‐NRGs in the testing set. (A) Boxplots showing the expression levels of 13 sFlu‐NRGs between the sFlu and non‐sFlu groups. (B) Violin plot showing the distribution of immune cells between the sFlu and non‐sFlu groups. (C) The connection between immune cell infiltration and 13 sFlu‐NRGs. Figure S4: Identification of NET‐related molecular clusters in severe influenza. (A) Consensus clustering matrix when *k* = 2. (B) CDF delta area curves. (C) The score of consensus clustering. (D) T‐SNE visualization of the distribution of the two clusters. Figure S5: Network diagram showing that the DGIdb database predicts 5 hub NRGs related to drugs.

## Data Availability

The data that support the findings of this study are available in the Supporting Information of this article.
