# Supplementary figures and images for: Neutrophil Extracellular Trap‐Related Gene Signatures and Molecular Clusters in Severe Influenza: Identification Through Integrative Transcriptome Analysis
Source: J Immunol Res. 2026 Feb 7;2026:6671885. doi: 10.1155/jimr/6671885 (PMC13140821; doi:10.1155/jimr/6671885)

Before batch correction

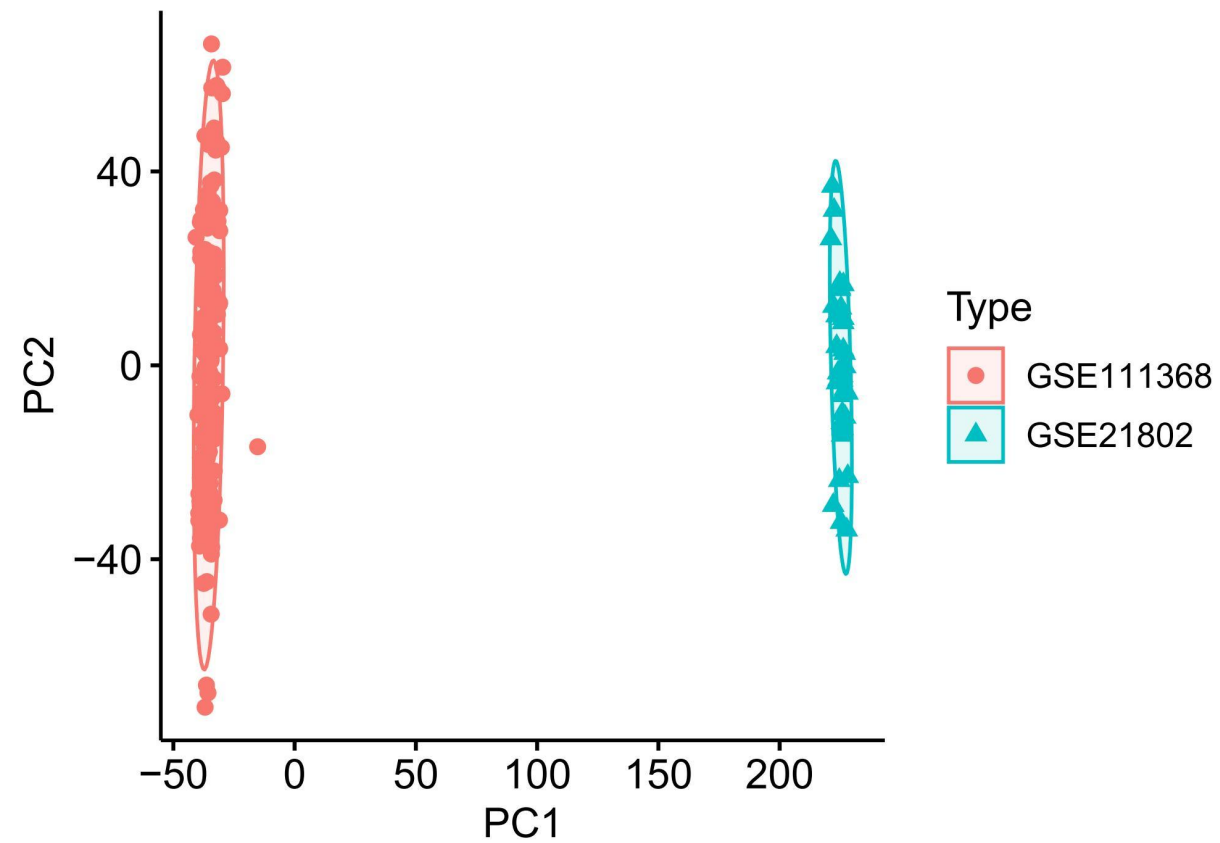

(A)

After batch correction

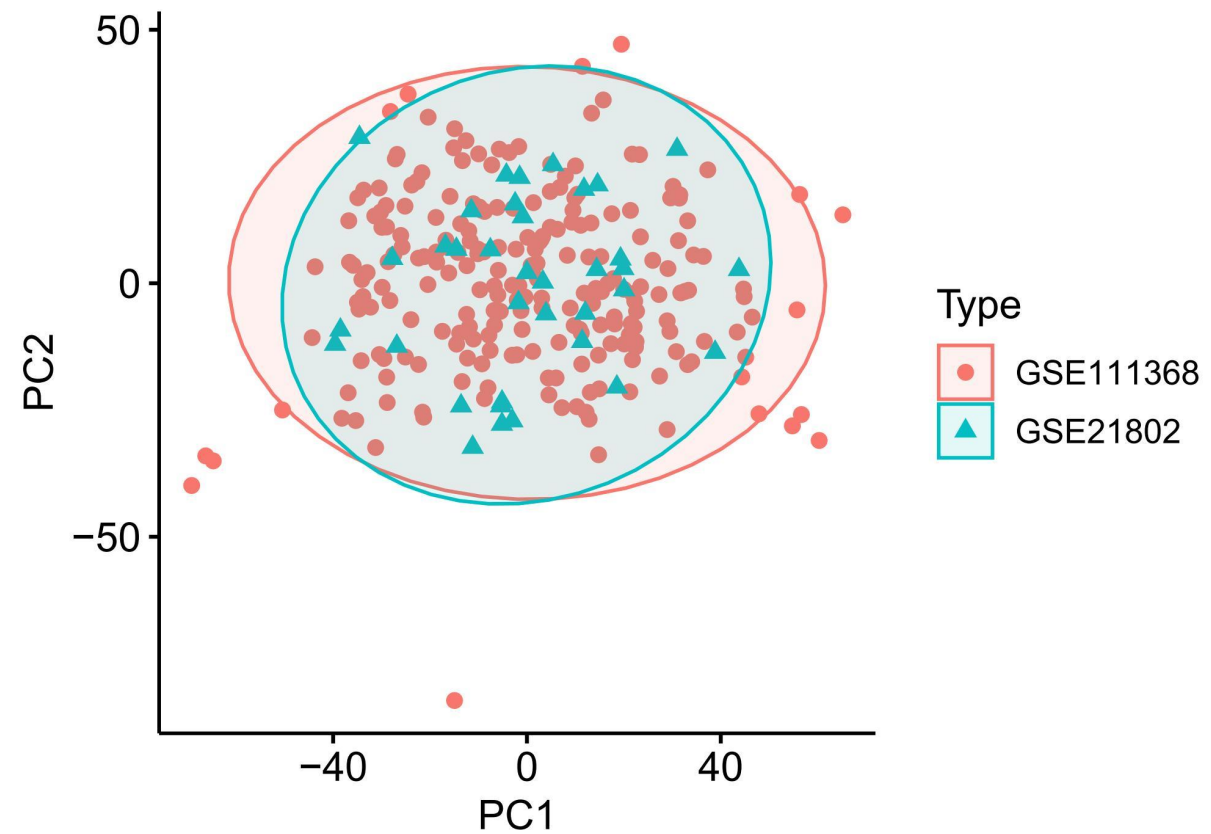

(B)

Fig S1

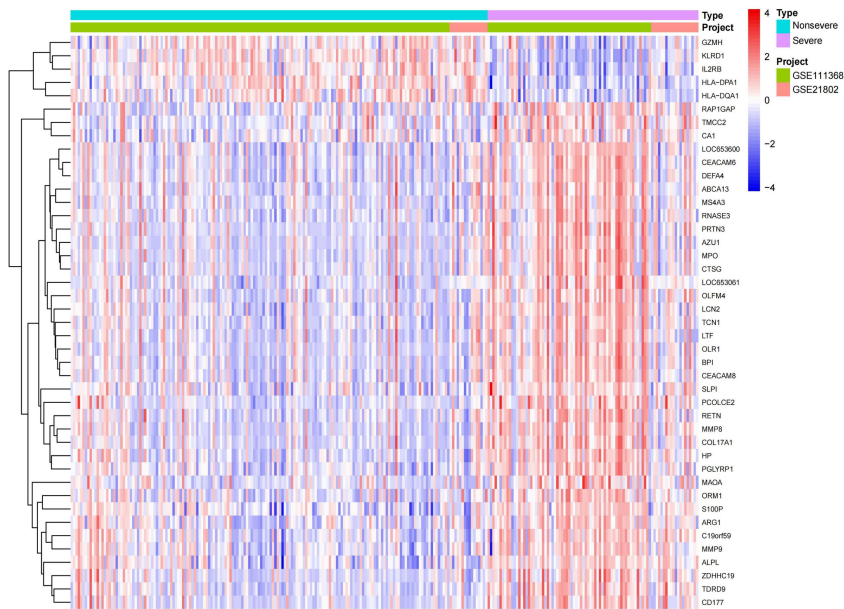

(A)

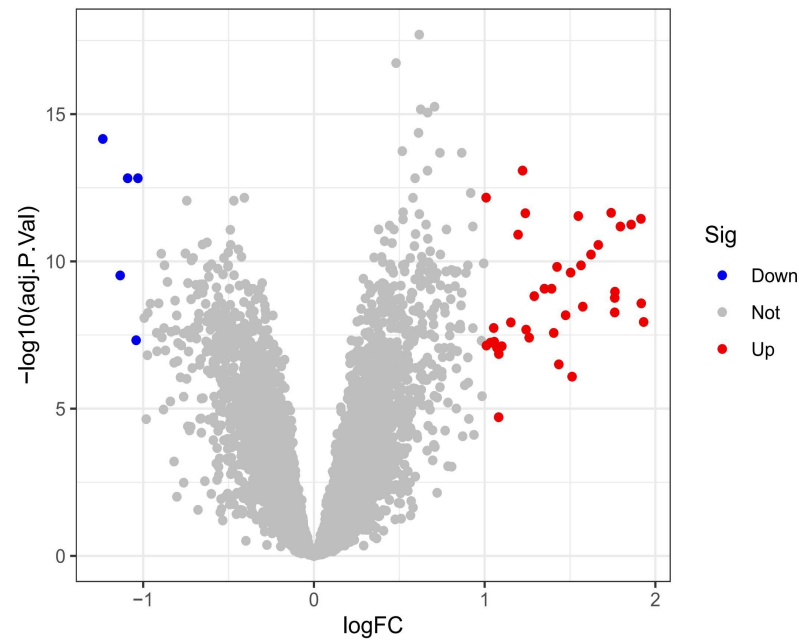

(B)

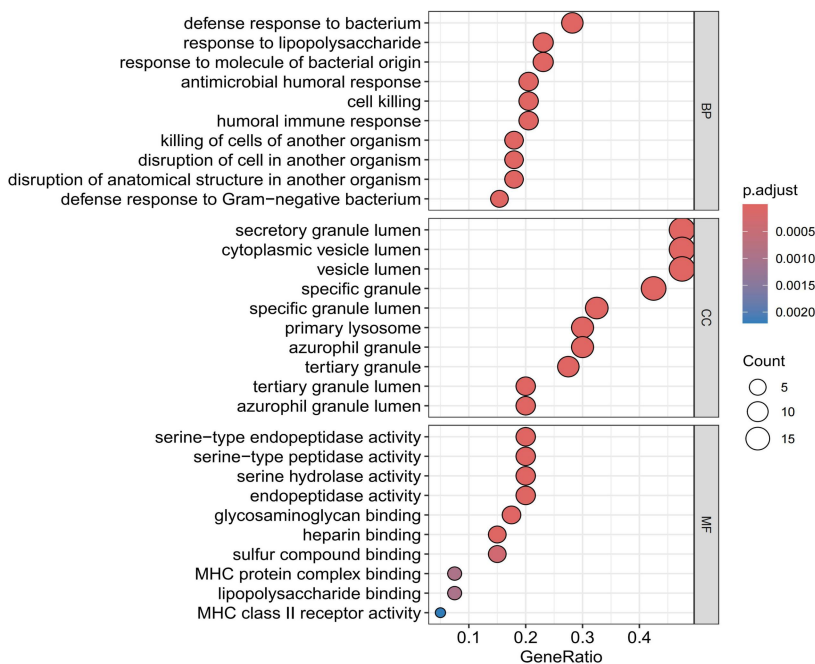

(C)

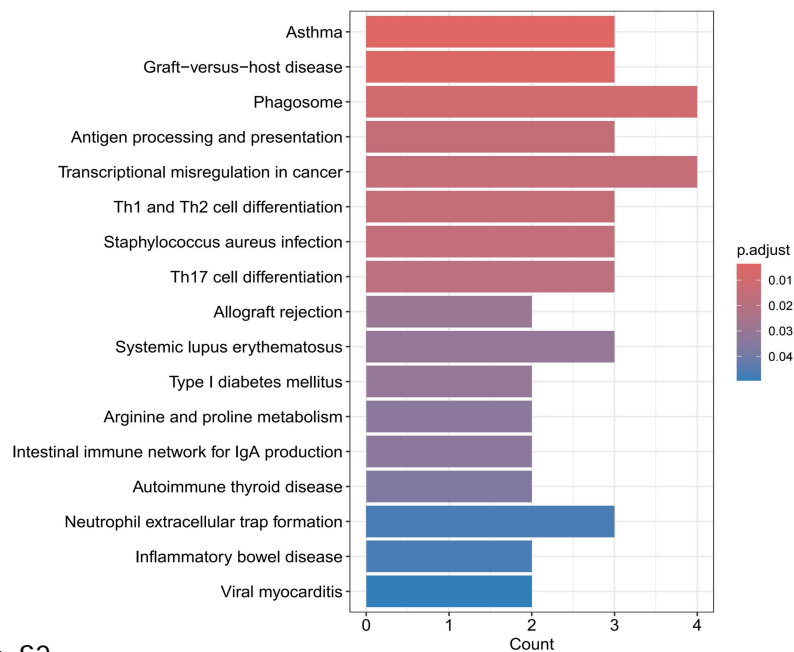

(D)

Fig S2

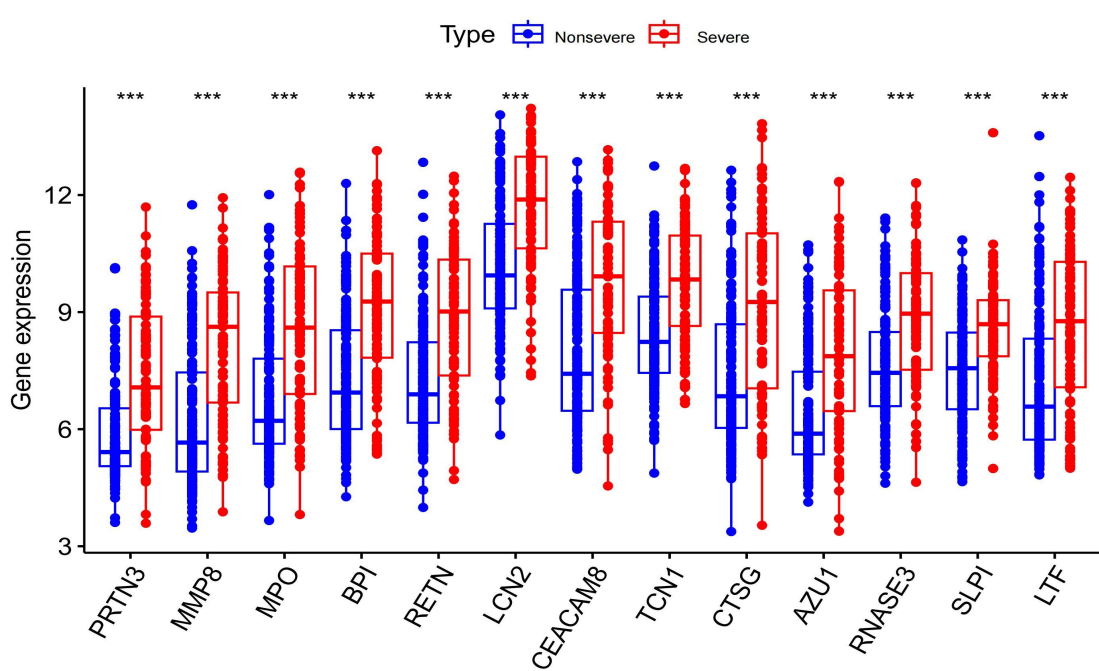

(A)

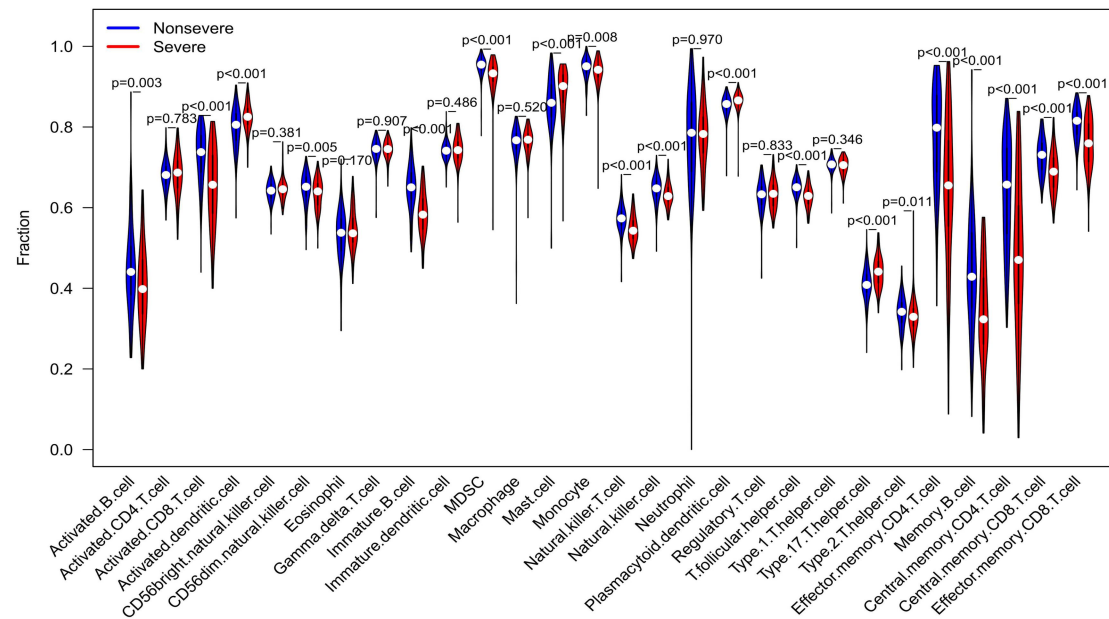

(B)

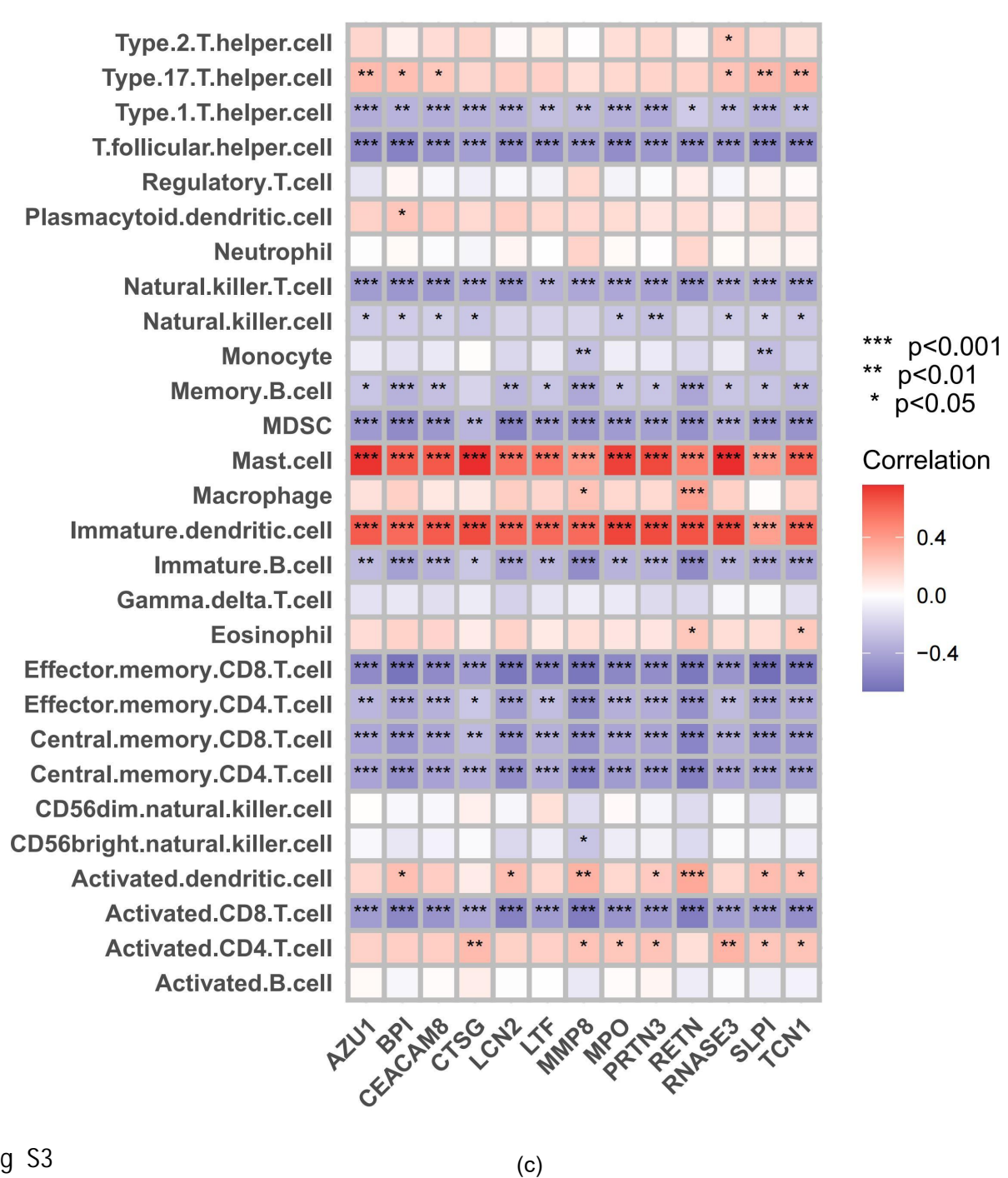

Fig S3

(c)

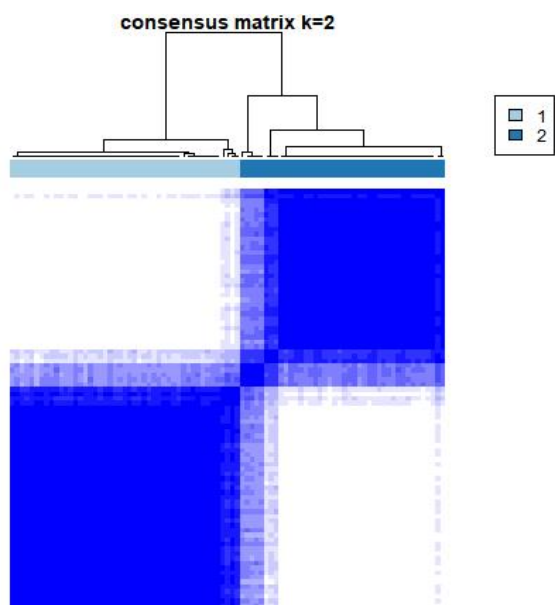

(A)

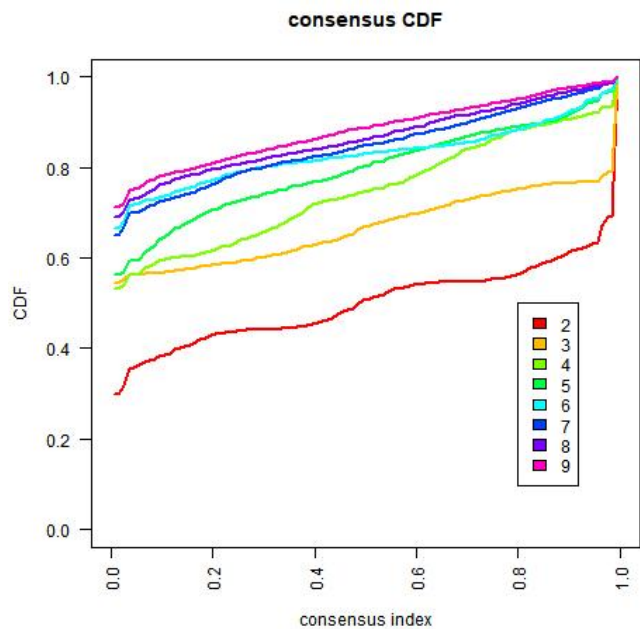

(B)

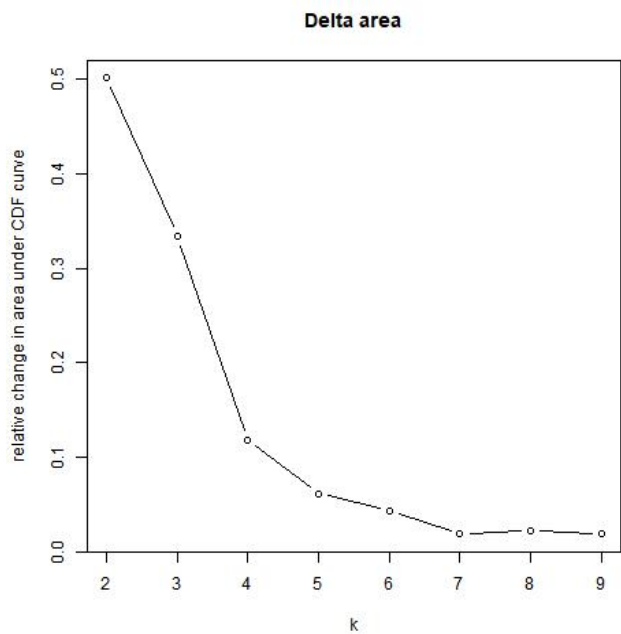

(C)

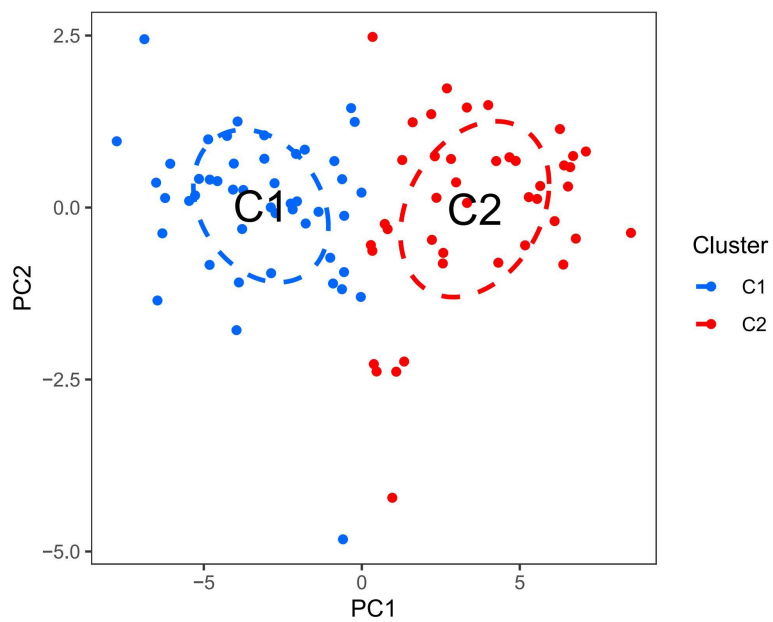

(D)

Fig S4

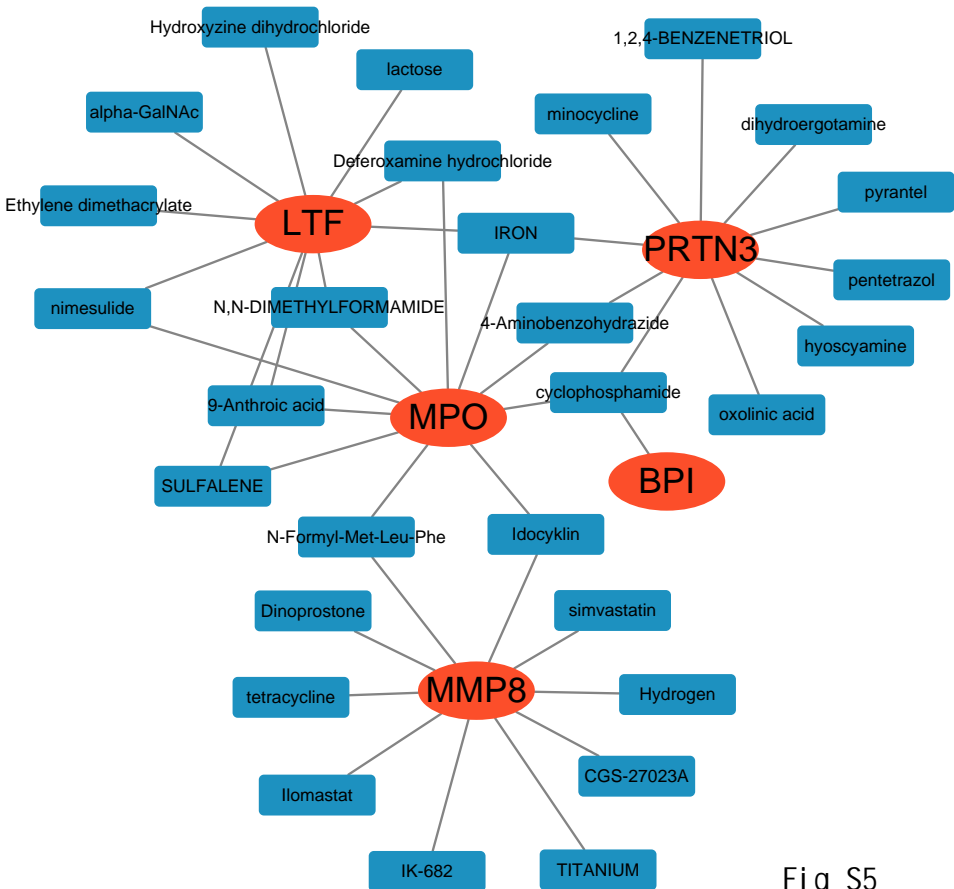

Fig S5

Supplement: Supplementary file 6 — Supporting Information 6 Figure S1: Principal component analysis of the gene expression dataset. The dots in the scatter plot are based on the first two main components of the gene expression profile (PC1 and PC2) visualization samples: (A) no elimination of the batch effect; (B) after elimination of the batch effect. The colors represent samples from the two different datasets. Figure S2: Gene expression profiling and functional enrichment analysis in the training set. (A) Heatmap of the top 50 DEGs. The upregulated genes are shown in red, whereas the downregulated genes are highlighted in blue. (B) DEG volcanic plot. The upregulated genes are highlighted in red, whereas the downregulated genes are indicated in blue. GO (C) and KEGG (D) analyses of the DEGs. Figure S3: Expression profiling and ICI analysis of 13 sFlu‐NRGs in the testing set. (A) Boxplots showing the expression levels of 13 sFlu‐NRGs between the sFlu and non‐sFlu groups. (B) Violin plot showing the distribution of immune cells between the sFlu and non‐sFlu groups. (C) The connection between immune cell infiltration and 13 sFlu‐NRGs. Figure S4: Identification of NET‐related molecular clusters in severe influenza. (A) Consensus clustering matrix when k = 2. (B) CDF delta area curves. (C) The score of consensus clustering. (D) T‐SNE visualization of the distribution of the two clusters. Figure S5: Network diagram showing that the DGIdb database predicts 5 hub NRGs related to drugs. [file JIMR-2026-6671885-s006.pdf]
